# The Use of Medications and Dietary Supplements by Masters Athletes — a Review

**DOI:** 10.1007/s13668-022-00398-0

**Published:** 2022-05-30

**Authors:** Joanna Harnett, Mike Climstein, Joe Walsh, Janelle Gifford

**Affiliations:** 1 Nutritional Pharmacology, Sydney Pharmacy School, Faculty of Medicine and Health, The University of Sydney, Camperdown, NSW 2005 Australia; 2 Clinical Exercise Physiology, Faculty of Health, Southern Cross University, Bilinga, QLD 4225 Australia; 3 Physical Activity, Lifestyle, Ageing and Wellbeing Faculty Research Group, The University of Sydney, Camperdown, Australia; 4 Sport Science Institute, Sydney, NSW Australia; 5 Discipline of Exercise and Sport Science, Sydney School of Health Sciences, Faculty of Medicine and Health, The University of Sydney, Camperdown, NSW 2005 Australia; 6 Sport and Physical Activity Research and Teaching Network, The University of Sydney, Sydney, Australia

**Keywords:** Masters athletes, Dietary supplements, Drug-nutrient interactions, Chronic conditions, Sports nutrition, Medications

## Abstract

***Purpose of Review*:**

Masters athletes (MA) are generally considered healthier than their sedentary peers. However, the prevalence of chronic conditions in any population increases with age. Treatments involve pharmacological and non-pharmacological interventions. A substantial proportion of the general population also use dietary supplements (DS). This raises questions about the potential for drug-nutrient interactions which may lead to adverse effects. We sought to determine the potential for drug-nutrient interactions MA may be exposed to by examining the prevalence of chronic conditions treated with medications and their DS use.

***Recent Findings*:**

Common conditions in MA include hypertension, hyperlipidemia, asthma, osteoarthritis, depression and anxiety. Treatments may involve prescribed medications. Few recent studies were identified on DS use; however, indications are for around 60% prevalence of supplement usage.

***Summary*:**

The higher prevalence of DS use by MA may result in drug-nutrient interactions that impact the effectiveness and safety of prescribed medications for chronic conditions.

## Introduction

Masters athletes (MA) are older individuals who exceed the recommended guidelines for physical activity or are involved in competition and/or systematic training [1]. In competition, the minimum age requirement is usually decided by each sport’s governing body [2], although they are generally over 35 years of age. Along with age, other characteristics such as level of activity and competitive goals (recreational through to internationally competitive), health and demographic characteristics vary widely. While MA are generally reported to be in better health than their sedentary peers [3, 4], physiological changes commonly associated with increasing age [5] and the increased incidence of chronic health conditions [6] may influence their overall health profile.

Masters athletes have been proposed by a number of investigators to be a model of successful ageing [3, 4, 7–9]. This is primarily based upon the well-documented benefits associated with physical activity and exercise on reduced all-cause cardiovascular mortality [10–12] and cause-specific mortality [13]. There is strong support in the literature for the effect of physical activity and exercise in reducing the risk for chronic disease and mortality, while also providing a means for primary chronic disease prevention, typically through mitigation of risk factors [11]. It is therefore presumable that life-long participation in physical activity and exercise is beneficial in preventing a number of chronic diseases and conditions, as well as premature death [14]. Specific to MA, health benefits have been documented with regard to a number of biomedical health determinants and risk factors [15] for chronic disease including obesity via lowering body mass index [4, 16–21], lipids [22], resting blood pressure [23] and improving glycemic control [24]. However, there has been no large-scale, longitudinal study which has investigated whether MA are immune from developing chronic diseases and conditions that are commonly associated with ageing and how they are managed.

The possibility of taking prescribed medications for a chronic condition along with the prevalent use of dietary supplements (DS) in the sporting environment may expose MA to adverse effects due to drug-nutrient interaction(s). Due to the ‘light touch’ regulatory frameworks in many countries, including the USA and Australia, access to DS products does not require consultation or interaction with a healthcare professional, and these products can be easily accessed through pharmacy, retail and online outlets [25, 26]. Concerns about potential drug-nutrient and drug-herb interactions have been reported [27]. Therefore, initiatives that encourage disclosure about prescribed medicine and DS use to healthcare professionals are needed to prevent adverse interactions from occurring [28–30].

This review sought to determine the prevalence of chronic conditions in MA that may involve treatment with prescribed medicines, and the types of DS used by MA as reported in the literature in the last decade. This is followed by a discussion of the possible nutrient-drug interactions to which MA may be exposed.

## Chronic Conditions in Masters Athletes

**Fig. 1:**
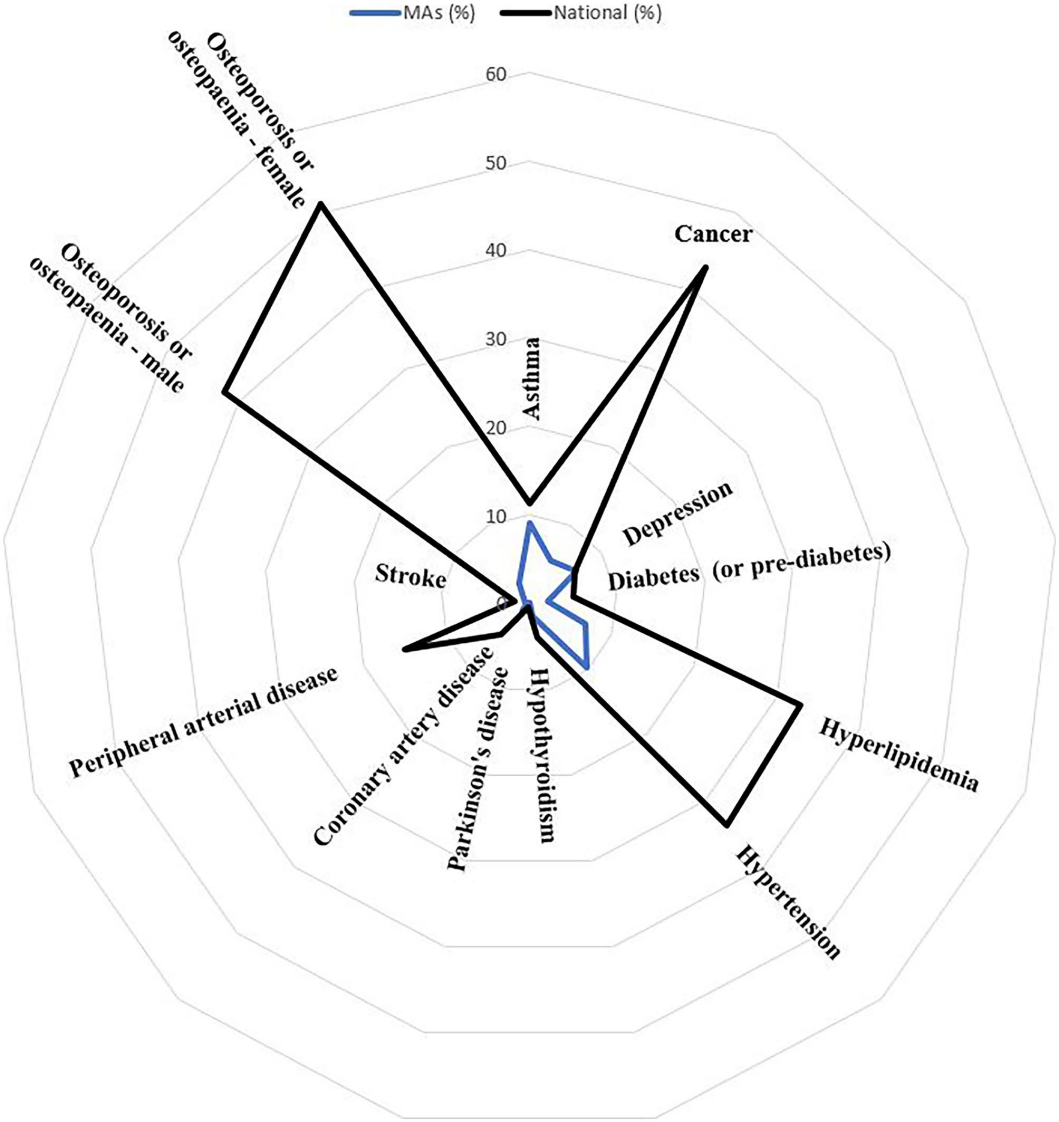
Radar plot of prevalence of chronic conditions in MA participating in the 2009 World Masters Games as compared to the Australian general population (modified with permission from reference [32•])

In the last decade, there have been limited studies on MA and their prevalence of chronic disease. Those available, primarily surveys, have investigated chronic disease and conditions in MA participating at sporting competitions. DeBeliso and colleagues [31] surveyed over 900 North American World Masters Games (WMG) participants and reported that MA had a lower prevalence of a number of chronic conditions as compared to the US general population including hypertension (9.1% vs 33%; *p* < 0.01), rheumatoid/osteoarthritis (10% vs 33%; *p* < 0.01), hypercholesterolemia (8.0% vs 6.7%; *p* < 0.01), asthma (6.5% vs 8.2%; *p* < 0.01) and depression (5.5% vs 8.0%; *p* < 0.01). Climstein et al. [32•] investigated the medical and health histories of over 8000 MA participating at the World Masters Games and compared their results to the Australian general population [33]. They reported a lower prevalence of asthma, coronary artery disease, stroke, cancer (all types combined), depression, diabetes (impaired fasting glucose, impaired glucose tolerance, type 1 and type 2, combined), hypercholesterolemia, hypertension, hypothyroidism, osteoporosis/osteopenia, Parkinson’s disease and peripheral arterial disease in MA as compared to the Australian general population (Fig. 1).

With regard to subsets of MA, Climstein and colleagues [34] recruited approximately 3200 veteran rugby players (aged 35 to 80 years) participating in an international masters rugby competition to investigate the prevalence of chronic conditions. Compared to the Australian general population [35], MA had a lower prevalence of anxiety (0.0% vs 3.0%; *p* < 0.05) and depression (3.2% vs 6.0%; *p* < 0.05). Halar and colleagues combined results from 817 MA surveyed at the Australian Masters Games (*n* = 130) and the Pan Pacific Masters Games (*n* = 687) and reported on the prevalence of chronic conditions compared to the Australian adult population [36•]. They found that MA had a lower age-gender-adjusted prevalence for cardiovascular conditions (11.7% vs 30.2%), hypertension (4.6% vs 18.7%), hyperlipidemia (1.6% vs 12.1%), type 2 diabetes mellitus (1.6% vs 5.5%), cancers (all types; 4.3% vs 11.9%), osteoporosis (1.9% vs 5.1%), asthma (9.9% vs 20.7%) and anxiety-related disorders (9.3% vs 18.1%). Kwon and colleagues [37] investigated cardiovascular related outcomes in MA (*n* = 50) as compared to age-matched healthy controls. The resting heart rate of MA was lower than controls (62.8 vs 74.0 bpm, *p* < 0.05) and MA had a lower incidence of pre-ventricular contractions during exercise (0.0% vs 24.0%; *p* < 0.001).

Despite limited research on chronic diseases and conditions in MA, it appears that MA may have a lower prevalence of several chronic conditions including hypertension, cardiovascular disease, hypercholesterolemia, cancer (all types combined), arthritis, osteoporosis, anxiety/depression, and asthma [32•, 36•, 38]. However, the potentially increased occurrence of coronary artery disease (CAD) in MA discussed below is unexpected in this context and not widely known. It is however recognised that the atherosclerotic process begins in early childhood to adolescence [39] and the long-term development of atherosclerosis may outweigh the beneficial effects of chronic physical activity and exercise. The atherosclerotic plaque development in middle-aged and older athletes has, however, been reported to be more stable (based upon plaque type) and hence associated with a lower cardiovascular morbidity and mortality [40].

### Coronary Artery Conditions in Masters Athletes: a Special Case

Although the findings from limited studies suggest MA have a lower prevalence for several chronic conditions, some studies have identified risk for CAD in this cohort. Dores et al. [41] investigated 195 MA deemed low to intermediate cardiovascular risk by risk factors, via coronary artery calcium scoring (CAC) and CT angiography. They reported that over one-fourth (25.7%) of the participants were identified with a high (i.e. CAC scores ≥ 75th percentile) atherosclerotic burden (multivessel disease, including the left main coronary artery and left anterior descending artery). Approximately 6% of the participants were identified with obstructive lesions. Additional testing by these clinical researchers [42] identified abnormal graded exercise tests (12.4%), electrocardiographic criteria for myocardial ischemia (5.7%) and exercise-induced ventricular arrhythmias (considered pre-lethal, 6.7%) in the MA.

Merghani and colleagues [43] assessed CAD in 152 MA and compared to age- and gender-matched sedentary controls. They reported MA had a higher prevalence of atherosclerotic plaque (*p* = 0.009), predominantly calcified (72.7%) versus mixed (calcified and non-calcified) plaque in the sedentary controls (61.5%). Of interest, the number of years MA trained was the only independent variable associated with plaque risk and luminal stenosis ≥ 50%. These investigators also conducted 24-h ECG monitoring on all participants and reported male MA had a higher incidence of atrial fibrillation (sixfold), supraventricular tachycardia (a pre-lethal arrhythmia, threefold) and non-sustained supraventricular tachycardia (non-lethal, ninefold).

## Dietary Supplement Use in Masters Athletes

There have been very few studies in the last decade reporting the prevalence of DS use in MA where data on MA can be separated out from other age groups. Guthrie et al. [44•] examined the prevalence of DS use in US Masters Swimming (USMS) members via an electronic survey and compared intake to the US population via data collected in the National Health and Nutrition Examination Survey (NHANES; 2007–2010). The authors hypothesised that MA would have a higher intake of DS than the general US population. Members were asked if they had taken DS in the previous 30 days. All supplements from both surveys were text entries with investigators manually extracting the data and grouping responses into nine categories with at least 10 respondents identifying usage. The USMS athletes (*n* = 1042) were older, more likely to be female and Caucasian, in better health, more educated and had a greater income than the NHANES sample. The prevalence of supplement use overall (62%) and all supplement categories (1.1–55.8%) was higher than the NHANES cohort in all instances except for ginkgo biloba. The most common supplements were DS used in the prevention or treatment of clinical conditions [45]: vitamins (55.8%), calcium supplements (23.1%), glucosamine and chondroitin (11.6%) and coenzyme Q10 (CoQ_10_; 5.4%). While the intake of the DS red rice yeast/extract by MA was lower than the general population, there are concerns about it interacting with statins as discussed below. Use of performance enhancing supplements was lower (DHEA/testosterone, 1.2%; creatine, 1.2%). While this study provides a useful insight into DS use by US masters swimmers, the authors acknowledge that the results cannot be generalised to all MA. Additionally, only data on those supplements that could be compared to the NHANES cohort was collected.

Evidence on DS use in athletes typically focusses on elite groups, however, is similarly estimated to be 40–100% [46]. Apart from the report from Guthrie et al. [44•], there are few recent studies specifically in MA that report the prevalence of supplement use. Graham-Paulson et al. reported that athletes over 41 years old with an impairment were most likely to take multivitamin and mineral supplements compared to younger groups. Specific data on different age categories could not be separated [47]. In contrast, Solheim and colleagues found that the use of non-ergogenic aids such as vitamins and minerals was lower in elite athletes and fitness customers aged 35–49 versus younger participants; however, there were only 8 participants reporting use in this age group compared to 215 younger participants [48]. Before 2010, Striegel et al. [49] reported an overall prevalence of DS use (60.5%) at the 2004 World Masters Athletic Championships (*n* = 598) similar to Guthrie et al. [44•]. The most common supplements reported were vitamins (35.4%) and minerals (29.9%), and creatine was used by 6.5% of those surveyed.

Importantly, reasons for use in MA appear to be different to elite athletes. Masters athletes may be more likely to use DS for health reasons (injury, 25.5%; other health reasons, 19.9%) rather than the performance-related reasons reported by elite groups [49]. This is consistent with findings in the fitness setting where older participants were more likely to use supplements for health and treating nutritional deficiencies, whereas younger participants were more likely to use ergogenic aids [50]. Data from the general population also indicates that the rate of consumption of special dietary foods such as protein powders was higher in young men than overall [51]. Recent evidence indicates that increased intake of protein [52, 53] (potentially via a supplement), creatine [54, 55], nitrates [56] (e.g. via beetroot juice) and other supplements [57] may be beneficial to the health of the ageing athlete and so in some cases, supplement intake may be encouraged by health professionals. Maters athletes’ concern for maintaining their health and increased risk of chronic conditions with age raises the possibility of concurrent use of prescribed medications and DS that may result in adverse side effects. Our observations from data collection at the 2017 Australian Masters Games (AMG) and the 2018 Pan Pacific Masters Games (PPMG) are that almost 50% of MA respondents reported taking up to 16 DS and sports foods, and again, almost 50% used two or more therapies inclusive of medications to treat chronic conditions [58]. Although there were no data collected on concurrent administration, use for treatment of conditions suggests this is the case.

While the focus of this review is not on aspects of the legality of medication and supplement taking in sport, it should be noted that the possibility of consuming a banned substance is also a consideration for MA. They are bound by the International Masters Games Association Anti-Doping rules [59] and may be tested for doping violations. We have observed that many athletes competing at masters games are relatively inexperienced (e.g. ≤ 5 years’ competitive experience) and compete for social or recreational reasons, while others participate for competitive reasons. Inexperienced MA may be unaware of adverse health effects of supplements as well anti-doping regulations in sport. Due to the social nature of many masters competitions, the use of alcohol in this context is an issue that is seldom raised but needs to be discussed. Additionally, we know anecdotally that more experienced competitors may risk adverse health effects including interactions between ingested substances (supplements or medications) that are potentially banned to enhance performance; to our knowledge, it is unreported in the literature. These athletes have often competed when younger and usually know that testing at masters level competitions occurs less frequently than in elite settings, effectively ‘gaming the system’. This activity should be brought to light and addressed at an administrative level. Education on these potentially fatal activities may assist in better understanding their consequences.

## Potential Interactions Between Commonly Used Medicines for Chronic Conditions, and Dietary Supplements Used by Masters Athletes

The conditions experienced by MA including osteoarthritis, hypertension, hyperlipidemia depression, anxiety and asthma, albeit significantly lower than in the general population[36•] are typically managed with a combination of non-pharmacological (dietary and lifestyle) and pharmacological interventions [60]. Dietary supplements are also used for these conditions [44•]. A study exploring a representative sample of the USA population and involving 820 individuals reported one in every two people who were taking specific medications to treat infection and/or cardiovascular risk factors (tetracyclines, thiazides or angiotensin II receptor blockers) were at risk of an interaction with a DS [61•]. This section discusses terms around drug-herb-nutrient interactions followed by potential interactions between DS and medications likely to be taken by some MA to treat chronic conditions identified in this review.

### Causes of a Drug-Herb-Nutrient Interaction

An interaction between a prescribed medication and a DS ingredient (herb or nutrient) can occur due to an alteration in the pharmacokinetic mechanisms and/or pharmacodynamic properties caused by concurrent intake [62]. A pharmacokinetic interaction is described as enhanced or reduced absorption, distribution, metabolism and excretion of the drug that occurs when two substances are combined [63]. A pharmacodynamic interaction refers to the alteration of a drug’s pharmacological effect and mechanism of action caused by the combination of two or more substances [27]. The clinical consequences of pharmacokinetic and pharmacodynamic interactions vary depending upon whether the effects of the drug are reduced or increased [64]. Increases in a drug’s activity can potentiate its pharmacological activity and increase unwanted side effects and toxicity profiles [64]. Reductions in a drug’s pharmacological activity can result in a sub-optimal therapeutic outcome [64]. Furthermore, interactions between drugs and nutrients may result in a compromised nutritional status [62, 63]. While not an extensive coverage of all potential drug-nutrient interactions, the following seeks to discuss some of the interactions that may be relevant to MA within the context of the conditions they have, medications used to treat those conditions and DS used.

### Glucosamine and Chondroitin Interactions with Medicines Used in the Management of Cardiovascular Disease

The available evidence indicates that glucosamine and chondroitin are commonly used among MA for osteoarthritis pain and disability [44•]. However, the evidence regarding the effectiveness of glucosamine and chondroitin for osteoarthritis suggests any statistical trends towards a benefit are of questionable clinical significance [65]. Case report data suggests a pharmacodynamic interaction is possible when glucosamine and chondroitin are taken concurrently with warfarin [66, 67]. Warfarin is an anticoagulant medication commonly used in the prevention of blood clots in patients with atrial fibrillation, myocardial infarction and those with prosthetic heart valves. The potentiation of warfarin’s effect by glucosamine and chondroitin is evidenced by an increase in the International Normalisation Ratio (INR) and a resultant risk of bleeding [66, 67]. When considering the risk–benefit ratio, collectively the minimal clinical benefits of glucosamine and chondroitin in managing the symptoms of osteoarthritis and the potential for a moderately severe adverse interaction, it would be considered prudent to avoid this combination.

### Ginkgo biloba and Interactions with Medicines Used in Cardiovascular Disease, Anxiety and Depression

Gingko biloba is a herb promoted for use in a range of chronic conditions including ‘improving circulation, and cognition and memory’ [68]. The use of gingko biloba supplements was also reported by Guthrie et al. to be higher among MA included in their study than the general population [44•]. Caution has been advised about concurrent use with gingko biloba in patients taking anticoagulant drugs such as warfarin, and drugs with anti-platelet effects such as aspirin and ticlopidine. This caution is based upon gingko biloba’s inhibitory effect on platelet aggregation as shown in both in vitro and animal studies and case reports of spontaneous and/or protracted bleeding [69, 70]. However, evidence obtained from studies evaluating the herb’s impact on coagulation status in healthy people have not identified any alterations to bleeding or pharmacokinetic and pharmacodynamic measurements of warfarin [71], or other anticoagulant or antiplatelet medications [64]. One study reported a potential interaction associated with the concurrent use of ginko biloba and the local anaesthetic agent bupivacaine [72]; the patient experienced a retrobulbar haemorrhage. 

Increased risk of perioperative bleeding during hip and knee arthroplasty in people taking ginkgo biloba and selective serotonin reuptake inhibitor (SSRI) antidepressant medications has been reported [73]. Both gastrointestinal tract bleeding and intracranial bleeding have also been associated with SSRI use [74]. Therefore, the theoretical concerns and associations with increased risk of bleeding, and potentiation of SSRI pharmacological effects from ginkgo biloba warrant caution until further evidence is obtained.

### Coenzyme Q10 and Interactions with Medicines Used to Treat Cardiovascular Risk Factors

Guthrie et al reported MA use of coenzyme Q_10_ (CoQ_10_) was substantially higher than the general population [44•]. Coenzyme Q_10_ is commonly used for its antioxidant and mitochondrial bioenergetic properties [75]. When considering drug interactions, theoretically, due to the structural similarity of CoQ_10_ and vitamin K, theoretically it is possible that concurrent use may reduce the effectiveness of warfarin’s anticoagulant effects [75]. Conversely and theoretically, the effect of CoQ_10_ effects on platelet size and receptors may increase the risk of bleeding in patients taking antiplatelet drugs including aspirin [76].

Regarding statin use, studies have reported significant reductions in serum CoQ_10_ associated with statin therapy for hyperlipidaemia [77–81]. Statins lower serum CoQ_10_ by inhibiting the enzyme 3-hydro-3-methylglutaryl Coenzyme-A (HMG-CoA) reductase, a rate-limiting step of cholesterol biosynthesis, which subsequently inhibits the conversion of mevalonate to CoQ_10_ [82]. Collectively these reductions in serum CoQ_10_ levels, coupled with an understanding the role of CoQ_10_ in mitochondrial energy production, have prompted the hypothesis that statin-induced CoQ_10_ deficiency is causal of statin-associated muscle symptoms [83]. Interestingly, to date, no studies have investigated a causal relationship between statin-associated muscle symptoms and CoQ_10_ depletion which may explain the conflicting results between reviews of clinical studies reporting on the efficacy of CoQ_10_ in treating this side effect [84, 85].Within the context of hypertension and diabetes, CoQ_10_ has been shown to have hypotensive [86] and antioxidant effects [86]. Therefore, monitoring blood pressure and blood glucose levels may be prudent during the early stage of concurrent use of CoQ_10_ and conventional antihypertensives and/or medications used to manage diabetes [86, 87].

### Red Yeast Rice Extract and Interactions with Statins Used to Treat Hyperlipidaemia

Promotion of red yeast rice extract’s cholesterol lowering proprieties makes it one of the most commonly used DS in Asia and Europe [88], and may be used by MA [44•]. Red yeast rice contains chemicals that are similar to statin medications including monacolin K, which has the same chemical structure as the lipid lowering drug lovastatin [88]. Red yeast rice has shown to be effective in reducing cholesterol via its naturally occurring constituent ‘lovastatin’ [89]. Studies investigating the pharmacokinetic properties of pure lovastatin and red yeast rice extracts have been conducted to examine the potential for drug interactions of either forms [90]. They identified red rice extracts containing the lovastatin-like compounds inhibited several of the drug metabolising cytochrome P450 enzymes and drug transporter P-glycoprotein activity more than pure lovastatin [90, 91]. In addition, the concomitant administration of red yeast rice extract with medications that are strong inhibitors of the P450 enzyme CYP3A4 could theoretically increase plasma levels of monacolin K, and therefore reduce the safety of red yeast and increase its statin like side effects [92]. A recent review on the safety of red rice extract concluded that despite the overall lack of interaction data associated with red yeast rice extract, patients should be monitored when taking other medications, especially those that are considered to be at a higher risk of statin-associated muscle symptoms [92].

### Calcium

While not a common condition reported by MA in this review, Halar et al reported that MA > 50 years old are more likely to experience poorer bone health (osteoporosis or osteopaenia) than younger MA [36•]. Osteoporosis is often treated with agents that affect bone metabolism including bisphosphonates [93]. Bisphosphonates have been shown to form insoluble complexes with multivalent cations such as calcium, aluminium magnesium and iron [93]. Therefore, co-administration of these minerals as DS and/or antacid medication may have a deleterious effect on the bioavailability of oral bisphosphonates [93, 94]. Co-administration of calcium is also known to interact with some antibiotics (quinolones and tetracyclines) by forming insoluble quinolone/tetracycline-calcium complexes, thereby reducing the absorption of the antibiotics [95]. Separation of calcium and these antibiotics by a minimum of 2 h may minimise this interaction and prevent sub-optimal therapeutic outcomes.

### Creatine and Caffeine

The use of creatine supplements has been reported to be higher in MA than in the general population [44•]. Creatine is an amino acid derivative that plays a role in creatine kinase, cellular energy provision and intracellular energy shuttling and is used widely in sports recovery and performance [96]. More recently, it has been suggested to have a positive influence on brain function [55]. Little is known about potential interactions between creatine and drugs used to treat common conditions of MA. Interestingly, higher caffeine intake when used concurrently with creatine supplementation in people living and medicated for Parkinson disease has been associated with a significantly faster progression of the disease that is attributed to a negative synergistic effect [97]. Caffeine alone did not have this effect [97].

## Conclusion

The literature is scarce regarding the prevalence of chronic conditions and DS use in the broader MA population. However, what is known is that MA do experience osteoarthritis, hypertension, hyperlipidemia, depression, anxiety and asthma, albeit lower than the general population. The available evidence to support pharmacodynamic and pharmacokinetic interactions between the medications used to treat these conditions and the DS used by MA suggests caution is warranted and monitoring required. In some cases, avoiding concurrent use is prudent to avoid harm. Importantly, further research is needed in many instances to establish stronger causal associations and build the evidence base. Furthermore, initiatives that promote awareness about potential drug-nutrient-herb interactions are encouraged and encouraging disclosure about DS to health professionals are encouraged. Masters athletes should be carefully counselled by sports physicians, sports dietitians, general practitioners and pharmacists about concurrent use of prescribed medications and DS. MA are encouraged to disclose their use. Health professionals advising MA on health and sporting performance should be proactive in seeking information from MA about medication and DS use as well as other co-occurring practices such as use of banned substances and alcohol which could potentially be dangerous.
